# Oncological Outcomes After Robotic Salvage Radical Prostatectomy in Patients Primarily Treated With Focal Versus Radiation Therapy: A Junior ERUS/YAU Collaborative Study

**DOI:** 10.1002/pros.70020

**Published:** 2025-07-23

**Authors:** Mike Wenzel, Arjun Nathan, Marcio Covas Moschovas, Christian Wagner, Giorgio Calleris, Fabrizio Di Maida, Juan Gomez Rivas, Carlo Andrea Bravi, Ruben De Groote, Federico Piramide, Filippo Turri, Keith Kowalczyk, Christoph Würnschimmel, Gopal Sharma, Iulia Andras, Edward Lambert, Nikolaos Liakos, Danny Darlington, Marco Paciotti, Gabriele Sorce, Philipp Mandel, Antonio Galfano, Senthil Nathan, Giancarlo Marra, Paolo Dell'Oglio, Alexandre Mottrie, Felix K. H. Chun, Vipul Patel, Alberto Breda, Alessandro Larcher

**Affiliations:** ^1^ Department of Urology Goethe University Hospital Frankfurt Frankfurt am Main Germany; ^2^ University College London Hospitals NHS Foundation Trust London UK; ^3^ AdventHealth Global Robotics Institute Celebration Florida USA; ^4^ Prostate Center Northwest, Department of Urology, Pediatric Urology and Uro Oncology St. Antonius‐Hospital Gronau Germany; ^5^ Urology Clinic, Department of Surgical Sciences University of Turin and Città della Salute e della Scienza Turin Italy; ^6^ Department of Experimental and Clinical Medicine University of Florence ‐ Unit of Oncologic Minimally‐Invasive Urology and Andrology, Careggi Hospital Florence Italy; ^7^ Department of Urology Hospital Clínico San Carlos Madrid Spain; ^8^ Department of Urology The Royal Marsden NHS Foundation Trust London UK; ^9^ Department of Urology Onze‐Lieve‐Vrouwziekenhuis Hospital Aalst Belgium; ^10^ ORSI Academy Ghent Belgium; ^11^ Division of Urology, Department of Oncology, San Luigi Gonzaga Hospital University of Turin Turin Italy; ^12^ Department of Urology, ASST Santi Paolo e Carlo University of Milan Milan Italy; ^13^ Department of Urology MedStar Georgetown University Hospital Washington District of Columbia USA; ^14^ Department of Urology Luzerner Kantonsspital Lucerne Switzerland; ^15^ Department of Urologic Oncology Medanta The Medicity Gurgaon India; ^16^ Department of Urology Iuliu Hatieganu University of Medicine and Pharmacy Cluj‐Napoca Romania; ^17^ Department of Urology Ghent University Hospital Ghent Belgium; ^18^ Department of Urology, Faculty of Medicine Medical Centre of the University of Freiburg Freiburg Germany; ^19^ Department of Urology, Stokes Centre for Urology Royal Surrey County Hospital Guildford UK; ^20^ Department of Urology Humanitas Research Hospital‐ IRCCS Rozzano Italy; ^21^ Department of Urology IRCCS San Raffaele Hospital Milan Italy; ^22^ ASST Grande Ospedale Metropolitano Niguarda Urology Department Milan Italy; ^23^ Department of Urology, Universitat Autònoma de Barcelona Fundació Puigvert Barcelona Spain

**Keywords:** BCR, brachytherapy, MFS, recurrent prostate cancer, SRARP

## Abstract

**Background:**

To evaluate surgical and cancer‐control outcome differences in robotic salvage radical prostatectomy (s‐RARP) patients after primary prostate cancer treatment with radiation (RT) versus focal therapy (FT).

**Methods:**

The Junior ERUS/Young Academic Urologist Working Group Robotics in Urology conducted a multicentric project to investigate biochemical recurrence‐free (BCR), metastases‐free (MFS) and overall survival outcomes in s‐RARP patients primarily treated with RT versus FT.

**Results:**

Overall, 439 s‐RARP patients qualified for analyses, of which 54% initially received RT with a median time interval between primary cancer treatment and s‐RARP of 48 months. Patients with RT more frequently exhibited unfavorable oncological characteristics before s‐RARP (PSA, ISUP score), as well as pathological ISUP score (all ≤ 0.01), relative to FT patients. No differences in postoperative complications were observed (*p* > 0.9). In BCR‐free analyses, no significant differences between RT and FT were observed (hazard ratio [HR]: 1.30, *p* = 0.2). In MFS and OS analyses, patients with RT harbored a higher risk of metastases (HR: 10.1, *p* < 0.001) and death (HR: 5.1, *p* = 0.02), relative to FT s‐RARP patients, but not after multivariable adjustment. In subgroup analyses of 201 FT patients, 80% received high‐intensified focused ultrasound (HIFU). No difference in BCR‐free survival was observed for HIFU‐ versus non‐HIFU s‐RARP patients (*p* = 0.9).

**Conclusions:**

Important differences in tumor characteristics between RT versus FT s‐RARP patients exist. These baseline differences translate into unfavorable short‐term MFS‐ and OS outcomes for RT s‐RARP patients.

## 1 Introduction

For patients with recurrent prostate cancer after initial curative treatment, salvage radical prostatectomy remains a potentially curative option in selected cases, offering acceptable oncological outcomes [1, 2]. However, due to its unfavorable functional results and considerable perioperative morbidity—particularly following initial radiation therapy—sRP remains a rarely performed procedure and is recommended only in high‐volume, experienced centers [1, 3, 4, 5].

With global advances in surgical techniques over the past decade and the widespread adoption of robot‐assisted procedures, outcomes of salvage radical prostatectomy are currently under renewed scientific scrutiny [6, 7, 8, 9, 10]. Moreover, with improvements and more frequent usage of focal therapy for primary prostate cancer treatment, relapsing prostate cancer is no longer a phenomenon after initial primary radiation therapy. With relapsing rates up between 32% and 89% after initial focal therapy, clinicians may be more frequently confronted with decision‐making towards a possible salvage radical prostatectomy [11, 12].

To the best of our knowledge, little is known about surgical, as well as cancer‐control outcomes, such as biochemical‐recurrence free (BCR), metastases‐free (MFS) and overall survival (OS) after salvage radical prostatectomy, stratified according to initial treatment of radiation therapy versus focal therapy [13, 14, 15]. Moreover, due to the rarity of the procedure, most reports focused on small sized patient cohorts and could therefore not focus on robotically performed salvage radical prostatectomies [16].

We addressed this void and conducted a multi‐center study within high volume robotic prostate cancer centers across the world and within the Junior ERUS/Young Academic Urologist Working Group Robotics in Urology. We hypothesized that clinically important surgical differences, as well as cancer‐control outcomes, namely BCR, MFS, and OS rates, may be observed, when robotic salvage radical prostatectomy (s‐RARP) patients are compared, stratified according to initial treatment with either radiation therapy versus focal therapy. Moreover, we hypothesized additional differences between s‐RARP patients undergoing different focal therapies may exist.

## 2 Materials and Methods

### 2.1 Study Population

After approval of the local ethics committee at the primary investigator's center (number: SUG‐5‐2018) and in accordance with the Declaration of Helsinki, we conducted a multicenter study of patient who underwent s‐RARP between 2008 and 2023 after initial prostate cancer treatment with either radiation therapy or focal therapy (Table S1). Exclusion criteria consisted of patients with unknown initial treatment, metastatic disease at recurrence, as well as castration‐resistant prostate cancer. After applying these criteria, 439 s‐RARP patients from 13 surgical centers worldwide were included.

### 2.2 Data Collection and Oncological Outcomes

All retrospectively collected data such as baseline patient and tumor characteristics, as well as surgical characteristics or oncological outcomes of relapsing s‐RARP patients were anonymously sampled. BCR was defined as a PSA rise to ≥ 0.2 ng/mL following s‐RARP. Patients with PSA persistence after s‐RARP were excluded from BCR analyses. MFS was defined as the time from surgery to the first occurrence of non‐regional metastasis. OS was defined as the time from surgery until death from any cause.

### 2.3 Statistical Analysis

Descriptive statistics included frequencies and proportions for categorical variables. Medians and interquartile ranges (IQR) were reported for all continuous variables. The Chi‐square test was used access differences in proportions. In addition, the *t*‐test and Kruskal‐Wallis tests were used to compare distributions of continuous variables. Postoperative complications were classified according to Clavien Dindo criteria.

For estimated annual percentage changes (EAPC) between primary radiation versus focal therapy over the last 10 years log‐linear regression models were used.

Kaplan‐Meier curves were used to graphically depict BCR, MFS, and OS outcomes in patients with initial radiation therapy versus focal therapy. Additionally, univariable and multivariable Cox regression models were used to adjust for potential confounders between groups as thoroughly as possible. In multivariable models, adjustments were made for PSA before s‐RARP, age at surgery, time interval between initial primary prostate cancer treatment and surgery for relapsing prostate cancer, positive surgical margins, pathological stage after surgery and pathological Gleason/ISUP score.

In subsequent analyses, patients with initial focal therapy were stratified abased on the use of high‐intensity focused ultrasound (HIFU) versus non‐HIFU techniques. All tests were two sided with a level of significance set at *p* < 0.05. R software environment for statistical computing and graphics (version 3.4.3) was used for all analyses.

## 3 Results

### 3.1 Baseline Characteristics of s‐RARP Patients

Overall, 439 s‐RARP patients qualified for descriptive analyses (Table 1), of whom 54% received radiation therapy versus 46% focal therapy as primary prostate cancer treatment.

**Table 1:** Characteristics of 439 robotic salvage radical prostatectomy (sRP) patients stratified according to initial prostate cancer (PCa) treatment with either focal therapy (FT) versus radiation therapy (RT).

Characteristic	*N*	Overall, *N* = 439^1^	FT, *N* = 201 (46%)^1^	RT, *N* = 238 (54%)^1^	*p* value^2^
Follow up, months	407	24 (10, 48)	19 (8, 36)	30 (12, 66)	< 0.001
Month between initial PCa and sRP	367	48 (24, 77)	36 (20, 60)	66 (29, 101)	**< 0.001**
Age initial PCa	182	64 (59, 68)	65 (60, 69)	62 (58, 67)	0.056
Age sRP	372	68 (63, 72)	68 (64, 72)	68 (63, 72)	0.6
BMI	406	27.6 (25.3, 30.0)	27.3 (25.0, 29.5)	28.0 (25.7, 31.4)	**0.035**
CCI initial PCa	200	0 (0, 1)	0 (0, 1)	0 (0, 3)	0.054
CCI sRP	201	3 (2, 4)	3 (2, 4)	3 (2, 4)	0.8
PSA initial PCa	120	7 (6, 10)	6 (5, 7)	8 (6, 13)	**0.001**
PSA prior sRP	391	4.4 (2.7, 7.5)	5.1 (3.3, 9.2)	3.9 (2.5, 6.6)	**0.001**
ADT	388	118 (30%)	10 (5.5%)	108 (52%)	**< 0.001**
LND performed	439	273 (62%)	101 (50%)	172 (72%)	**< 0.001**
LND number	266	7 (3, 13)	8 (3, 13)	7 (3, 12)	> 0.9
EBL	403	100 (100, 250)	200 (100, 300)	100 (100, 200)	**< 0.001**
OR time	434	145 (120, 180)	150 (120, 180)	140 (120, 180)	0.4
Catheter days	177	10 (5, 21)	5 (3, 10)	14 (9, 28)	**< 0.001**
ECOG 1–2	51	20 (39%)	2 (12%)	18 (53%)	**0.005**
cT 3–4 sRP	359	89 (25%)	44 (25%)	45 (25%)	> 0.9
ISUP score at initial PCa	115				0.8
1		53 (46%)	19 (50%)	34 (44%)	
2		28 (24%)	10 (26%)	18 (23%)	
3		17 (15%)	6 (16%)	11 (14%)	
4		7 (6.1%)	1 (2.6%)	6 (7.8%)	
5		10 (8.7%)	2 (5.3%)	8 (10%)	
ISUP score at prior sRP	410				**< 0.001**
1		56 (14%)	22 (11%)	34 (16%)	
2		139 (34%)	92 (46%)	47 (22%)	
3		94 (23%)	42 (21%)	52 (25%)	
4		59 (14%)	21 (11%)	38 (18%)	
5		62 (15%)	22 (11%)	40 (19%)	
Uni‐/bilateral nerve‐sparing	438	249 (57%)	116 (58%)	133 (56%)	0.7
pT 3–4	434	246 (57%)	106 (53%)	140 (60%)	0.2
pN1	413	45 (11%)	20 (11%)	25 (11%)	0.8
PSM	435	133 (31%)	71 (36%)	62 (26%)	**0.040**
Pathological ISUP score	313				**< 0.001**
1		12 (3.8%)	5 (3.3%)	7 (4.3%)	
2		91 (29%)	72 (48%)	19 (12%)	
3		77 (25%)	39 (26%)	38 (23%)	
4		30 (9.6%)	8 (5.3%)	22 (14%)	
5		103 (33%)	27 (18%)	76 (47%)	
Complications CD grade	66				> 0.9
1		43 (65%)	14 (70%)	29 (63%)	
2		12 (18%)	3 (15%)	9 (20%)	
3		5 (7.6%)	2 (10%)	3 (6.5%)	
3a		3 (4.5%)	1 (5.0%)	2 (4.3%)	
3b		2 (3.0%)	0 (0%)	2 (4.3%)	
4a		1 (1.5%)	0 (0%)	1 (2.2%)	
EAU criteria fulfilled	190	52 (27%)	20 (24%)	32 (30%)	0.3
PSA persistence	433	40 (9.2%)	17 (8.5%)	23 (9.9%)	0.6
RT					
EBRT				128 (54%)	
Brachytherapy				99 (42%)	
Brachytherapy + EBRT				11 (4.6)	
FT	201				
Cryotherapy		30 (15%)	30 (15%)		
HIFU		160 (80%)	160 (80%)		
IRE		9 (4.5%)	9 (4.5%)		
PDT		1 (0.5%)	1 (0.5%)		
TULSA		1 (0.5%)	1 (0.5%)		

*Note:* Bold values indicate statistically significant.

Abbreviations: ADT, androgen deprivation therapy; BMI, Body mass index; CCI, Charlson Comorbidity Index; CD, Clavien‐Dindo; EBL, estimated blood loss; EBRT, External Beam Radiation Therapy; ECOG, Easter Cooperative Oncology Group; HIFU, high‐intensified focused ultrasound; IRE, irreversible electroporation; ISUP, International Society of Urological Pathology; LND, lymph node dissection; OR, operating room; PDT, photodynamic therapy; PSA, prostate‐specific antigen; PSM, positive surgical margin; TULSA, transurethral ultrasound ablation.

^1^ Median (IQR); *n* (%).

^2^ Kruskal‐Wallis rank sum test; Fisher's exact test; Pearson's Chi‐square test.

Median time interval between primary cancer treatment and s‐RARP was 48 months (IQR: 24–77) at a median age at surgery of 68 years (IQR: 63–72) with a PSA of 4.4 ng/mL (IQR: 2.7–7.5 ng/mL). Median follow‐up duration of the entire cohort was 24 months (IQR: 10–48 months).

### 3.2 Comparison of Radiation Versus Focal Therapy Patients

When comparing s‐RARP patients primarily treated with radiation versus focal therapy (Table 1), time interval between initial treatment and s‐RARP was significantly longer in radiation therapy patients (66 vs. 36 months, *p* < 0.001). Moreover, initial PSA level at prostate cancer diagnosis was higher (8.0 vs. 6.0 ng/mL), while PSA level before s‐RARP was lower in radiation therapy patients (3.9 vs. 5.1 ng/mL), relative to focal therapy patients (both *p* = 0.001). Moreover, the rate of ADT administration and rhe frequency of lymph node dissection were higher in radiation therapy patients (52% vs. 5.5% and 72% vs. 50%, both *p* < 0.001). The proportion of ISUP grade 4‐5 disease was higher both in pre‐salvage biopsy and in final pathology reports after s‐RARP among radiation therapy patients (37% vs. 22% and 61% vs. 23%, both *p* < 0.01). Conversely, positive surgical margin (PSM) rates were higher in focal therapy patients (36% vs. 26%, *p* = 0.04).

Regarding surgical complications and outcomes, no proportional differences were observed in accordance with Clavien Dindo classification (*p* > 0.9). Postoperative catheter‐dwelling time was significantly longer after initial radiation therapy (13 vs. 5 days, *p* < 0.001), while blood loss was higher in the focal therapy group (200 vs. 100 ml, *p* < 0.001).

**Figure 1:**
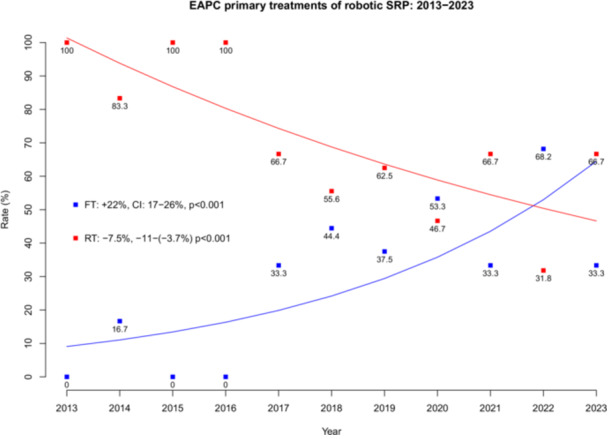
Estimated annual percentage changes (EAPC) within the last decade of robotic salvage radical prostatectomy (SRP) patients primarily treated with radiation therapy (RT) versus focal therapy (FT). [Color figure can be viewed at wileyonlinelibrary.com]

In time trend analyses over the last decade (Figure 1), the proportion of s‐RARP procedures following initial radiation therapy significantly decreased (EAPC: −7.5%, *p* < 0.001), while the proportion after focal therapy increased (EAPC: +22%, *p* < 0.001).

### 3.3 Oncological Outcomes of Radiation Versus Focal Therapy Patients

**Figure 2:**
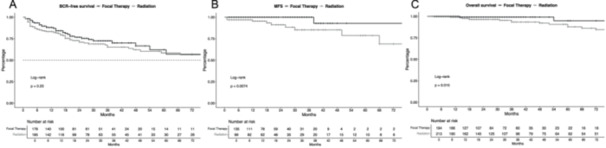
Kaplan Meier curves graphically depicting biochemical‐recurrence (BCR) free survival (A), metastasis‐free survival (MFS,B) and overall survival (C) of robotic salvage radical prostatectomy patients stratified according to primary therapy with radiation therapy versus focal therapy.

In BCR‐free analyses (Figure 2A), no significant differences between radiation therapy and focal therapy s‐RARP patients were observed with 55 versus 37 BCR events for the radiation therapy versus focal therapy group (hazard ratio [HR] for radiation therapy: 1.30, *p* = 0.23). 24‐ and 48‐month BCR rates were 71.0% and 62.1% for initial radiation versus 76.6% and 66.6% for initial focal therapy. After additional multivariable adjustment, no differences between both groups were observed (Table S2). Moreover, rates of PSA persistence after s‐RARP were comparable between both groups (radiation therapy 9.9% vs. focal therapy 8.5%, *p* = 0.6).

In MFS analyses (Figure 2B), patients with initial radiation therapy harbored a higher risk of metastases (HR: 10.1, *p* < 0.001), with 24‐ and 48‐month MFS rates of 91.2% and 85.3% versus 100% and 92.9% for radiation versus focal therapy. Metastatic events were 10 versus one for radiation versus focal therapy. After multivariable adjustment, no difference between both compared groups remained (Table S2, *p* = 0.5).

In OS analyses (Figure 2C), patients with initial radiation therapy also exhibited worse outcomes (HR: 5.1, *p* = 0.02), with 19 versus two deaths for radiation versus focal therapy. 24‐ and 48‐months OS rates were 96.3% and 92.1% versus 99.1% and 99.1% for radiation versus focal therapy. After multivariable adjustment, also no difference between both compared groups was observed (Table S2, *p* = 0.8).

### 3.4 Comparison of HIFU Versus Non‐HIFU Patients

Of 201 focal therapy patients, 80% initially received HIFU as primary treatment. Most common non‐HIFU focal therapy was cryotherapy. Descriptive baseline, surgical and postoperative comparisons between HIFU‐ versus non‐HIFU are displayed in Table 2.

**Table 2:** Characteristics of 201 robotic salvage radical prostatectomy (sRP) patients stratified according to initial prostate cancer (PCa) treatment with high‐intensified focused ultrasound (HIFU) versus other focal therapies (non‐HIFU).

Characteristic	*N*	Overall, *N* = 201^1^	HIFU, *N* = 160 (80%)^1^	Non‐HIFU *N* = 41 (20%)^1^	*p* value^2^
Month between initial PCa and sRP	198	36 (20, 60)	34 (19, 59)	45 (25, 66)	**0.041**
Age initial PCa	125	65 (60, 69)	65 (60, 69)	67 (63, 67)	0.9
Age sRP	166	68 (64, 72)	67 (63, 72)	69 (65, 72)	0.4
BMI	197	27.3 (25.0, 29.5)	27.3 (25.0, 29.5)	27.3 (24.9, 29.5)	0.7
CCI initial PCa	124	0 (0, 1)	0 (0, 1)	0 (0, 0)	0.3
CCI sRP	57	3 (2, 4)	4 (2, 4)	3 (2, 4)	0.4
PSA initial PCa	40	6.3 (5.3, 7.3)	6.3 (5.0, 7.2)	7.1 (5.8, 7.9)	0.5
PSA prior sRP	183	5.1 (3.3, 9.2)	5.0 (3.4, 8.4)	6.1 (2.6, 11.5)	0.8
LND performed	201	101 (50%)	68 (43%)	33 (80%)	**< 0.001**
LND number	79	0 (0, 0)	0 (0, 0)	0 (0, 1)	**0.023**
EBL	197	200 (100, 300)	200 (100, 300)	100 (88, 163)	**< 0.001**
OR time	201	150 (120, 180)	160 (120, 180)	134 (106, 150)	**0.002**
Catheter days	49	5 (3, 10)	6 (4, 10)	3 (2, 4)	0.070
ECOG 1–2	17	2 (12%)	2 (13%)	0 (0%)	> 0.9
cT 3–4 sRP	176	44 (25%)	38 (27%)	6 (18%)	0.3
ISUP at initial PCa	38				0.4
1		19 (50%)	17 (50%)	2 (50%)	
2		10 (26%)	9 (26%)	1 (25%)	
3		6 (16%)	6 (18%)	0 (0%)	
4		1 (2.6%)	1 (2.9%)	0 (0%)	
5		2 (5.3%)	1 (2.9%)	1 (25%)	
ISUP at prior sRP	199				**< 0.01**
1		22 (11%)	17 (11%)	5 (12%)	
2		92 (46%)	82 (52%)	10 (24%)	
3		42 (21%)	32 (20%)	10 (24%)	
4		21 (11%)	15 (9.5%)	6 (15%)	
5		22 (11%)	12 (7.6%)	10 (24%)	
ADT	182	10 (5.5%)	4 (2.7%)	6 (17%)	**0.005**
Uni‐/bilateral nerve‐sparing	201	116 (58%)	80 (50%)	36 (88%)	**< 0.001**
pT 3–4	200	106 (53%)	82 (52%)	24 (59%)	0.4
pN1	190	20 (11%)	12 (8.0%)	8 (20%)	**0.040**
PSM	200	71 (36%)	57 (36%)	14 (35%)	> 0.9
Pathological ISUP score	151				**< 0.001**
1		5 (3.3%)	4 (3.2%)	1 (3.8%)	
2		72 (48%)	67 (54%)	5 (19%)	
3		39 (26%)	35 (28%)	4 (15%)	
4		8 (5.3%)	5 (4.0%)	3 (12%)	
5		27 (18%)	14 (11%)	13 (50%)	
Complications CD grade	20				0.3
1		14 (70%)	12 (71%)	2 (67%)	
2		3 (15%)	3 (18%)	0 (0%)	
3		2 (10%)	2 (12%)	0 (0%)	
3a		1 (5.0%)	0 (0%)	1 (33%)	
EAU criteria fulfilled	85	20 (24%)	19 (28%)	1 (5.9%)	0.062
PSA persistence	201	17 (8.5%)	12 (7.5%)	5 (12%)	0.3

*Note:* Bold values indicate statistically significant.

Abbreviations: ADT, androgen deprivation therapy; BMI, Body mass index; CCI, Charlson Cormorbidity Index; CD, Clavien‐Dindo; EBL, estimated blood loss; ECOG, Easter Cooperative Oncology Group; ISUP, International Society of Urological Pathology; LND, lymph node dissection; OR, operating room; PSA, prostate‐specific antigen; PSM, positive surgical margin.

^1^ Median (IQR); *n* (%).

^2^ Kruskal‐Wallis rank sum test; Fisher's exact test; Pearson's Chi‐square test.

**Figure 3:**
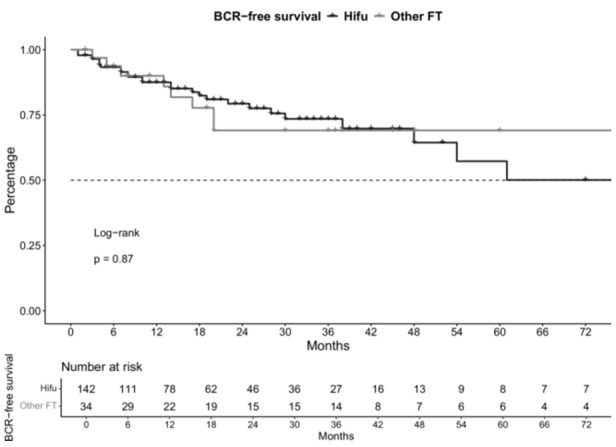
Kaplan Meier curve graphically depicting biochemical‐recurrence (BCR) free survival of robotic salvage radical prostatectomy patients stratified according to primary therapy with high‐intensity focused ultrasound (HIFU) versus other focal therapy (FT) techniques.

In BCR‐analyses (Figure 3), no significant difference was observed between patients with initial HIFU versus non‐HIFU (HR: 0.9, *p* = 0.9) with 29 versus 8 BCR events. 24‐ and 48‐months BCR‐free survival rates were 79.3% and 64.4% versus 69.1% and 69.1% for HIFU versus non‐HIFU s‐RARP patients.

## 4 Discussion

We conducted a multi‐center study within high volume robotic prostate cancer centers across the world and within the Junior ERUS/Young Academic Urologist Working Group on Robotics in Urology to address contemporary knowledge gaps in robotic salvage radical prostatectomy outcomes. We initially hypothesized that clinically meaningful differences in BCR‐, MFS‐, and OS rates may exist in s‐RARP patients according to initial prostate cancer treatment with either radiation therapy or focal therapy. We tested these hypotheses and made several important observations.

First, we observed statistically significant and clinically meaningful differences in patients who received initial radiation versus focal therapy. For example, median time between initial prostate cancer treatment and s‐RARP was substantially longer for radiation therapy patients (66 vs. 36 months). These observations are in agreement with previously published literature on focal therapies [17, 18]. For example a prospective trial found 50% recurrence rates after 3 years of follow up, i, other multicenter studies report rates of 31% failure‐free survival after 7 years of follow up [12, 19]. As a result, patients should be counseled in clinical practice regarding possible short‐ and midterm outcomes of primary prostate cancer focal therapies and the possibility of the need of salvage treatment.

Moreover, when characteristics between patents with radiation versus focal therapy with subsequent s‐RARP were compared, patients with initial radiation therapy more frequently harbored unfavorable tumor characteristics, such as higher PSA level at initial prostate cancer diagnosis or ISUP score 4–5 proportions before surgery. In this scenario, it may partially explain the less favorable oncological outcomes observed in radiation therapy patients in our study. These observations are not surprising and in line with previous comparisons, since radiation therapy, together with radical prostatectomy, is the standard of care for all risk categories of localized prostate cancer, while focal therapy indications are still under investigation and is mostly administered for low risk and favorable intermediate risk prostate cancer patients [1, 14, 20, 21, 22].

Although some have suggested that salvage radical prostatectomy might be less complex in patients after focal therapy versus radiation therapy, and despite longer time period until catheter removal in radiation therapy patients, no differences in postsurgical complication rates were observed and the majority of complications were classified as Clavien Dindo grade I–II (95% and 83%). These observations are also in an agreement with previously published literature. For example, Onol et al. also reported shorter time until catheter removal in FT patients [13]. Moreover, in the report by Bozkurt et al., relying on only robotically treated patients, 90% of complications were also attributable to Clavien Dindo grade I‐II. However, taking other studies with open surgical approaches into account, rates of grade ≥ III complications appear to be higher [23, 24]. Conversely, RT patients had longer Foley catheter durations, possibly reflecting delayed anastomotic healing due to radiation‐induced changes in the periprostatic tissues.

Second, when pathological features after surgery and cancer‐control outcomes were compared, also important observations were made. More specifically, patients with initial radiation therapy also harbored higher rates of ISUP score 4–5 proportions in final specimen, while PSM rates were significantly higher for focal therapy patients. However, the above‐described pre‐salvage and pathological disadvantages in radiation therapy patients did not translate in statistically significant or clinically meaningful BCR differences. However, the BCR‐free survival rates observed in our study cohort—62% for radiation therapy and 67% for focal therapy at 48 months—are comparable to those reported in other multicenter salvage radical prostatectomy cohorts [25]. Similarly, the two available studies comparing patients with prior radiation therapy versus focal therapy also reported no significant differences in BCR outcomes between the groups [13, 15]. However, it is of note that the initial longer time to salvage treatment in RT patients may have been influenced by the higher rates of ADT, compared to FT patients and may also affected cancer‐control outcomes.

Third, when MFS and OS outcomes were compared, significant survival disadvantages were observed for patients with initial treatment of radiation therapy. However, after adjusting for unfavorable characteristics in multivariable analyses, the MFS‐ and OS disadvantage for radiation therapy patients vanished. In consequence, it may be hypothesized that not the initial prostate cancer treatment determines cancer‐control outcomes, but rather the patient selection and their prostate cancer characteristics. However, rates of OS after 48 months remained high in the current cohort and ranged from 91% to 99% for radiation versus focal therapy patients and are also in an agreement with previously published large‐scale epidemiological or multicenter cohorts [26, 27].

Finally, stratification by type of focal therapy revealed that longer time to salvage treatment (45 vs. 34 months), less blood loss (100 vs. 200 mL) and less days until catheter removal (3 vs. 6), were observed for non‐HIFU patients versus HIFU patients. However, rates of complications and BCR‐rates remained comparable. To the best of our knowledge, no previous report focused on this specific topic. However, due to sample size and follow‐up limitations no further MFS and OS stratifications in this regard could be performed.

Moreover, further limitations should be acknowledged when the current study is interpreted. First, the retrospective design and potential differences in data sampling, clinical practices across different centers or unknown characteristics may limit outcomes, statistical power or may introduce some selection biases, similar as the number of included patients by some of the participating centers, specifically for some of the applied subgroup analyses. Moreover, no further data on quality of life or functional outcomes due to differences in sampling were unfortunately available [28]. However, differences in intraoperative settings or surgical techniques may influence outcomes, for which could not be adjusted for [29]. Nonetheless, the study was conducted with the intent to provide homogeneous data on robotically performed salvage radical prostatectomy patients and is currently the largest available cohort. However, further studies need to address more specific questions such as the influence of repeated focal therapies; one versus both sited focal therapy or different focal therapy modalities. Some of the provided variables may harbor inconsistencies in sampling and reporting such as baseline Gleason/ISUP score. Finally, data on new imaging stating modalities were unfortunately not available and may influence clinical decision making in the future in recurrent prostate cancer.

## 5 Conclusion

Taken together, our multicenter cohort from the Junior ERUS/Young Academic Urologist Working Group on Robotics in Urology demonstrates an increasing trend toward performing salvage RARP after initial focal therapy. Notably, the interval between primary and salvage treatment appears to be decreasing. Regarding oncological outcomes, patients with prior radiation therapy show disadvantages in MFS and OS compared to those initially treated with focal therapy—likely reflecting more adverse tumor characteristics in the radiation group.

## Ethics Statement

The current study was in accordance with the Declaration of Helsinki. Local Review of the ethics board was approved.

## Conflicts of Interest

The authors declare no conflicts of interest.

## Supporting information


**Supplemental Table 1:** Number of included patients per center, stratified by radiation therapy and focal therapy prior to salvage robotic radical prostatectomy.

**Supplemental Table 2:** Multivariable Cox regression models predicting biochemical recurrence (BCR), metastases‐free survival (MFS) and overall survival (OS) in robotic salvage radical prostatectomy patients stratified according to initial focal therapy vs. radiation therapy. Abbreviation: HR: Hazard Ratio, CI: Confidence interval.

## Data Availability

Data are available for bona fide researchers who request it from the authors.

## References

[1] N. Mottet , R. C. N. van den Bergh , E. Briers , et al. Guidelines on Prostate Cancer Published online (2019).

[2] P. Mandel , T. Steuber , S. Ahyai , et al., “Salvage Radical Prostatectomy for Recurrent Prostate Cancer: Verification of European Association of Urology Guideline Criteria,” BJU International 117, no. 1 (2016): 55–61, 10.1111/bju.13103. 25711672

[3] M. Wenzel , C. Würnschimmel , L. Nocera , et al., “Salvage Radical Prostatectomy: Baseline Prostate Cancer Characteristics and Survival Across SEER Registries,” Clinical Genitourinary Cancer 19, no. 4 (2021): 255, 10.1016/j.clgc.2021.03.015. 33849813

[4] J. Drobner , A. Kaldany , M. S. Shah , and S. Ghodoussipour , “The Role of Salvage Radical Prostatectomy in Patients With Radiation‐Resistant Prostate Cancer,” Cancers 15, no. 14 (2023): 3734, 10.3390/cancers15143734. 37509395 PMC10378204

[5] F. Blank , M. Meyer , H. Wang , et al., “Salvage Radical Prostatectomy After Primary Focal Ablative Therapy: A Systematic Review and Meta‐Analysis,” Cancers 15, no. 10 (2023): 2727, 10.3390/cancers15102727. 37345064 PMC10216462

[6] F. Preisser , C. Würnschimmel , R. M. Pose , et al., “Concordance of Biopsy and Pathologic ISUP Grading in Salvage Radical Prostatectomy Patients for Recurrent Prostate Cancer,” Prostate 82, no. 2 (2022): 254–259, 10.1002/pros.24268. 34807461

[7] M. Wenzel , C. Würnschimmel , L. Nocera , et al., “The Effect of Lymph Node Dissection on Cancer‐Specific Survival in Salvage Radical Prostatectomy Patients,” Prostate 81, no. 6 (2021): 339–346, 10.1002/pros.24112. 33666271

[8] M. C. Moschovas , C. A. Bravi , P. Dell'oglio , et al., “Outcomes of Salvage Robotic‐Assisted Radical Prostatectomy in the Last Decade: Systematic Review and Perspectives of Referral Centers,” International Brazilian Journal of Urology: Official Journal of the Brazilian Society of Urology 49, no. 6 (2023): 677–687, 10.1590/S1677-5538.IBJU.2023.0467. PMC10947626 37903005

[9] R. De Groote , A. Nathan , E. De Bleser , et al., “Techniques and Outcomes of Salvage Robot‐Assisted Radical Prostatectomy (sRARP),” European Urology 78, no. 6 (2020): 885–892, 10.1016/j.eururo.2020.05.003. 32461073

[10] K. J. Kowalczyk , R. H. Madi , C. G. Eden , et al., “Comparative Outcomes of Salvage Retzius‐Sparing Versus Standard Robotic Prostatectomy: An International, Multi‐Surgeon Series,” Journal of Urology 206, no. 5 (2021): 1184–1191, 10.1097/JU.0000000000001939. 34181471

[11] K. R. S. Bhat , A. Nathan , M. C. Moschovas , S. Nathan , and V. R. Patel , “Outcomes of Salvage Robot‐Assisted Radical Prostatectomy in Patients Who Had Primary Focal Versus Whole‐Gland Ablation: A Multicentric Study,” Journal of Robotic Surgery 17, no. 6 (2023): 2995–3003, 10.1007/s11701-023-01738-0. 37903973

[12] D. Reddy , M. Peters , T. T. Shah , et al., “Cancer Control Outcomes Following Focal Therapy Using High‐Intensity Focused Ultrasound in 1379 Men With Nonmetastatic Prostate Cancer: A Multi‐Institute 15‐Year Experience,” European Urology 81, no. 4 (2022): 407–413. 10.1016/j.eururo.2022.01.005. 35123819

[13] F. F. Onol , S. Bhat , M. Moschovas , et al., “Comparison of Outcomes of Salvage Robot‐Assisted Laparoscopic Prostatectomy for Post‐Primary Radiation vs Focal Therapy,” BJU International 125, no. 1 (2020): 103–111, 10.1111/bju.14900. 31430422

[14] E. Linares Espinós , R. Sánchez‐Salas , A. Sivaraman , et al., “Minimally Invasive Salvage Prostatectomy After Primary Radiation or Ablation Treatment,” Urology 94 (2016): 111–116, 10.1016/j.urology.2016.04.040. 27154045

[15] L. Ribeiro , T. Stonier , L. Stroman , et al., “Is the Toxicity of Salvage Prostatectomy Related to the Primary Prostate Cancer Therapy Received?,” Journal of Urology 205, no. 3 (2021): 791–799, 10.1097/JU.0000000000001382. 33021441

[16] P. Cathcart , L. Ribeiro , C. Moore , et al., “Outcomes of the RAFT Trial: Robotic Surgery After Focal Therapy,” BJU International 128, no. 4 (2021): 504–510. 10.1111/bju.15432. 33891378

[17] R. Nicoletti , A. Alberti , D. Castellani , et al., “Oncological Results and Cancer Control Definition in Focal Therapy for Prostate Cancer: A Systematic Review,” Prostate Cancer and Prostatic Diseases, 27, no. 4 (2024): 623–634, 10.1038/s41391-023-00699-7. 37507479

[18] S. Deivasigamani , S. Kotamarti , A. R. Rastinehad , et al., “Primary Whole‐Gland Ablation for the Treatment of Clinically Localized Prostate Cancer: A Focal Therapy Society Best Practice Statement,” European Urology 84, no. 6 (2023): 547–560, 10.1016/j.eururo.2023.06.013. 37419773

[19] B. Kaufmann , E. Raess , F. A. Schmid , et al., “Focal Therapy With High‐Intensity Focused Ultrasound for Prostate Cancer: 3‐Year Outcomes From a Prospective Trial,” BJU International 133, no. 4 (2024): 413–424, 10.1111/bju.16213. 37897088

[20] J. L. Mohler , E. S. Antonarakis , A. J. Armstrong , et al., “Prostate Cancer, Version 2.2019, NCCN Clinical Practice Guidelines in Oncology,” Journal of the National Comprehensive Cancer Network: JNCCN 17, no. 5 (2019): 479–505, 10.6004/jnccn.2019.0023. 31085757

[21] A. Borkowetz , A. Blana , D. Böhmer , et al., “German S3 Evidence‐Based Guidelines on Focal Therapy in Localized Prostate Cancer: The First Evidence‐Based Guidelines on Focal Therapy,” Urologia Internationalis 106, no. 5 (2022): 431–439, 10.1159/000521882. 35144260 PMC9153342

[22] M. Wenzel , H. Borgmann , J. Von Hardenberg , et al., “Acceptance, Indications and Chances Of Focal Therapy In Localized Prostate Cancer: A Real‐World Perspective Of Urologists in Germany,” Journal of Endourology 35, no. 4 (2021): 444–450. 10.1089/end.2020.0774. 32935562

[23] B. Devos , W. Al Hajj Obeid , C. Andrianne , et al., “Salvage High‐Intensity Focused Ultrasound Versus Salvage Radical Prostatectomy for Radiation‐Recurrent Prostate Cancer: A Comparative Study of Oncological, Functional, and Toxicity Outcomes,” World Journal of Urology 37, no. 8 (2019): 1507–1515, 10.1007/s00345-019-02640-x. 30666400

[24] A. Saouli , A. Ruffion , C. Dariane , et al., “Salvage Radical Prostatectomy for Recurrent Prostate Cancer: A Systematic Review (French ccAFU),” Cancers 15, no. 22 (2023): 5485, 10.3390/cancers15225485. 38001745 PMC10670522

[25] F. Preisser , R. B. Incesu , P. Rajwa , et al., “Oncologic Outcomes of Lymph Node Dissection at Salvage Radical Prostatectomy,” Cancers 15, no. 12 (2023): 3123, 10.3390/cancers15123123. 37370733 PMC10296518

[26] M. Wenzel , C. Würnschimmel , L. Nocera , et al., “The Effect of Race/Ethnicity on Cancer‐Specific Mortality After Salvage Radical Prostatectomy,” Frontiers in Oncology 12 (2022): 874945, 10.3389/fonc.2022.874945. 36059656 PMC9437357

[27] F. Preisser , R. B. Incesu , P. Rajwa , et al, “Impact of Persistent PSA After Salvage Radical Prostatectomy: A Multicenter Study,” Prostate Cancer and Prostatic Diseases 27, no. 4 (2024): 686–692, 10.1038/s41391-023-00728-5. PMC11543598 37803241

[28] S. Rodler , D. Danninger , L. Eismann , et al., “Health‐Related Quality of Life Following Salvage Radical Prostatectomy for Recurrent Prostate Cancer After Radiotherapy or Focal Therapy,” World Journal of Urology 42, no. 1 (2024): 242, 10.1007/s00345-024-04945-y. 38635030 PMC11026200

[29] S. Ferretti , P. Dell'oglio , D. Ciavarella , A. Galfano , L. Schips , and M. Marchioni , “Retzius‐Sparing Robotic‐Assisted Prostatectomy: Technical Challenges for Surgeons and Key Prospective Refinements,” Research and Reports in Urology 15 (2023): 541–552, 10.2147/RRU.S372803. 38106985 PMC10725648

