# Supplementary material for: Oncological Outcomes After Robotic Salvage Radical Prostatectomy in Patients Primarily Treated With Focal Versus Radiation Therapy: A Junior ERUS/YAU Collaborative Study
Source: Prostate. 2025 Jul 23;85(14):1332–41. doi: 10.1002/pros.70020 (PMC12379848; doi:10.1002/pros.70020)
Supplement: Supplementary file 1 — Supplemental Table 1: Number of included patients per center, stratified by radiation therapy and focal therapy prior to salvage robotic radical prostatectomy. [file PROS-85-1332-s002.docx]

| ****Center**** | Focal therapy | ****Radiation therapy**** |
| --- | --- | --- |
| Florence | 13 | 1 |
| Kissimmee | 41 | 94 |
| Frankfurt | 2 | 5 |
| Georgetown | 0 | 8 |
| Gronau | 21 | 32 |
| Gurgaon | 1 | 0 |
| London | 110 | 54 |
| Lucerne | 0 | 1 |
| Madrid | 0 | 12 |
| Milan | 1 | 2 |
| Ghent | 2 | 9 |
| San Luigi Gonzaga Turin | 8 | 0 |
| Città della Salute e della Scienza Turin | 2 | 20 |
