# Supplementary material for: Oncological Outcomes After Robotic Salvage Radical Prostatectomy in Patients Primarily Treated With Focal Versus Radiation Therapy: A Junior ERUS/YAU Collaborative Study
Source: Prostate. 2025 Jul 23;85(14):1332–41. doi: 10.1002/pros.70020 (PMC12379848; doi:10.1002/pros.70020)
Supplement: Supplementary file 2 — Supplemental Table 2: Multivariable Cox regression models predicting biochemical recurrence (BCR), metastases‐free survival (MFS) and overall survival (OS) in robotic salvage radical prostatectomy patients stratified according to initial focal therapy vs. radiation therapy. Abbreviation: HR: Hazard Ratio, CI: Confidence interval. [file PROS-85-1332-s001.docx]

|  | Multivariable analyses | | |
| --- | --- | --- | --- |
| BCR | **HR** | **CI** | **p value** |
| Focal therapy | **Ref.** | **-** | **-** |
| Radiation therapy | 0.89 | 0.46-1.73 | 0.7 |
| MFS | **HR** | **CI** | **p value** |
| Focal therapy | **Ref.** | **-** | **-** |
| Radiation therapy | 2.42 | 0.18-31.9 | 0.5 |
| OS | **HR** | **CI** | **p value** |
| Focal therapy | **Ref.** | **-** | **-** |
| Radiation therapy | 1.64 | 0.06-43.57 | 0.8 |
